# Comparison of Low- and High-Cost Infrared Thermal Imaging Devices for the Detection of Lameness in Dairy Cattle

**DOI:** 10.3390/vetsci9080414

**Published:** 2022-08-06

**Authors:** Aidan Coe, Nicola Blackie

**Affiliations:** Department of Pathobiology and Population Sciences, Royal Veterinary College, University of London, Hawkshead Lane, Hatfield AL9 7TA, UK

**Keywords:** infrared thermography, lameness, cattle, lameness detection, thermal imaging

## Abstract

**Simple Summary:**

Lameness has high economic and welfare cost to the U.K. dairy industry; accurate and early detection of lameness minimises this cost. Thermal imaging devices can be used as a method of detecting lameness; however, these devices are typically high-cost and fragile, limiting their usefulness in a farm setting. This study looked at the effectiveness of low-cost thermal imaging devices when used as lameness detection aids, by comparing one to a research-specification thermal imaging device. Thermal images were taken of cattle feet, and each cow was assessed for lameness. Both devices tested were able to determine whether the cattle were lame; however, the research-specification device performed marginally better at this function. This minimal difference in effectiveness between these devices suggests that low-cost thermal imaging devices could be used as a lameness detection aid; increased use of these devices by farmers may increase lameness detection rates and benefit animal welfare.

**Abstract:**

Lameness has a high economic cost to the U.K. dairy industry; accurate and early detection of lameness minimises this cost. Infrared thermal imaging (IRT) devices have shown promising results for use as a lameness detection aid in cattle when used in research settings; these devices are typically high-cost, limiting their adoption. This study analysed the effectiveness of low-cost IRT devices (LCDs) as lameness detection aids, by comparing both maximum environmentally adjusted temperature values and hindfeet temperature difference collected by an LCD to the mobility score of the cow; this test was repeated for data collected by a research-specification device. Data collection occurred during routine milking of 83 cattle; each cow’s mobility was scored afterwards. Significant differences were found between lame and sound cows with the LCD, upon analysis of both methods. There was no significant difference between the data captured by differing devices. The maximum sensitivity and specificity values for the LCD were calculated as 66.95 and 64.53, respectively, compared with 70.34 and 70.94, respectively, for the research-specification device; optimum threshold values for these were equivalent for both devices, suggesting IRT lameness identification is not device-dependent. It was concluded that a minimal difference in effectiveness between tested devices suggests that LCDs could be used as a lameness detection aid; consequently, there is potential for widespread adoption as on-farm detection aids.

## 1. Introduction

### 1.1. Lameness

Lameness is a clinical syndrome that interrupts normal gait with many predisposing risk factors [1]. In U.K. dairy herds, lameness is a very prevalent problem [2]; in 2020, the prevalence was estimated to be 29.5% in the United Kingdom [3], although the authors determined that this number varies between 13.8 and 48.2% [3]. Furthermore, farmers find it difficult to accurately assess lameness themselves, so robust lameness detection methods are crucial to tackling the problem [4]. The main causes of lameness in U.K. dairy cattle are claw lesions, with the hind claw involved in approximately 70% of cases [5]. The prevalence of lesion type varies between farms [6]; the most common lesions are digital dermatitis (DD) [7], white line disease (WLD) [8], and sole ulcers (SUs) [9,10]. DD is typically characterised by inflammation and erosion of the skin around the heel [11,12], whereas WLD and SU are present on the underside of the claw. Gold standard detection methods for these conditions involve visual inspection after lifting the foot; however, this is labour-intensive [13] and less invasive techniques are needed. Research suggests infrared thermography (IRT) has proven to be a reliable way of detecting, and potentially grading lesions [14], although this is dependent on the method used and lesion type; other factors, typically environmental, can have an impact on effectiveness, and IRT cannot diagnose SU or WLD without lifting of the foot [15]. 

### 1.2. Lameness Detection

Lameness identification is important in the dairy industry owing to high economic costs associated with the condition [16]. Lameness can often go undetected leading to delay in treatment [9] exacerbating the condition. Lameness detection systems are key to reduce both on-farm prevalence and economic cost [3]. Key requirements of these systems are to identify the lameness status of a cow and to determine which leg(s), if any, are affected. Currently, numerous methods are employed in lameness detection, varying in both cost and efficacy; the most commonly used method in the United Kingdom is mobility scoring [17], using the Agriculture and Horticulture Development Board (AHDB) four-point scale [3]. The advantages of using mobility score (MS) are the administration speed, low-cost, and low-labour requirements [18]; however, scoring is subjective, with only 72% of intra-observer repeatability and 37% inter-observer repeatability [19,20].

### 1.3. Thermography

Although IRT has been used in a variety of different functions in the veterinary and farming industries [21,22], owing to advances in IRT technology and its reported benefits, IRT is becoming more accessible and an increasingly attractive method of lameness detection [23]. IRT is a non-invasive procedure having no requirement for a lame animal to locomote [24]; provides instantaneous results of whether a cow is lame, requiring no period of data collection; and does not require any devices attached to the cow, making it a practical method of data collection at evaluating lameness in stationary animals, such as in the milking parlour [22]. Additionally, IRT allows the potential to detect lesions sooner than traditional methods as it allows for objective detection of lameness, rather than the subjective MS [25] and can provide better reliability of results than mobility scoring [26]. 

When measured with IRT, lame limbs have a higher temperature than unaffected limbs [25]. The likely mechanism behind this is more fully documented in an equine setting, but could be applicable to bovine [27]. Skin temperature is predominately affected by the blood supply to the area [28]; the greater the blood flow, the greater the surface temperature. For most acute causes of lameness, there is increased inflammation in the affected limb [29], leading to local vasodilation, with resultant increased blood flow and thus increased temperature [30]. It is suggested that this is not the same for chronic lameness cases [31]. In these, foot temperature may only be mildly raised, owing to ongoing inflammation reducing blood flow to the area by alternative mechanisms such as thrombosis, swelling, and tissue infarction [28]. Additionally, in both acute and chronic cases, the lame limb bears less weight, causing increased pressure in the other limbs; in chronic cases, this results in inflammation, increasing the temperature of those limbs [31]. 

Key specifications of IRT devices that affect the quality of thermograms produced are the resolution, thermal sensitivity, and accuracy [32,33]; the better these factors, typically the greater the cost of the camera [34,35]. Previous studies into cattle lameness using IRT devices have used research-orientated cameras with high specifications [25,26,36,37]. These cameras are expensive, up to GBP 20,000, and fragile, making on-farm use impractical and uneconomical [24,38,39,40,41]. Owing to technological advances, there are now low-cost IRT devices (LCDs) on the market, with comparatively lower specifications; these have not been tested for effectiveness in lameness detection [23], but have previously been determined to be adequate for some purposes, such as body temperature assessment at a close range [42]. 

There is no optimum method of IRT in cattle lameness detection, with no clear consensus on the ideal location from which to obtain thermograms, or on how to classify lame animals; multiple locations have been tested, including interdigital skin, coronary band, and heel bulbs [38,43]. The main trialed classification methods use a difference in temperature between pairs of feet, maximum temperature detected in a foot, or patterns of temperature characteristic of a lesion [25,26,43]. These methods vary in simplicity; measurement of the maximum temperature is simplest and favourable results have been found with this technique using various set threshold values [17,38,40,43]. These threshold values have varied, likely owing to differing methodologies and adjustment for external factors [25,44], preventing comparison of the study results and determination of an optimum temperature threshold to use. Uncontrollable factors have an impact on the temperature detected by the IRT device, either environmentally or cattle-dependent, and include ambient temperature, humidity, wind speed, and emissivity of the skin. Using the temperature difference between limbs may account for this, as the unaffected limb imaged acts as an intrinsic control for the individual animal [43,45].

Current barriers to widespread adoption of IRT for lameness detection include the high cost of IRT devices and difficulty implementing lameness detection programmes. Many farmers can use MS systems for their cattle for low-cost [46] and practical nature to obtain data, and the processing of the data from IRT devices is labour-intensive and difficult to carry out [43]. Additionally, IRT temperatures have been shown to vary between farms and have a low sensitivity to the detection of lameness [25,44], appearing to be more effective at identifying sound animals [44]. As a method, this is ineffective for national implementation; such an initiative would necessitate the collection of initial baseline data from each individual farm prior to lameness identification. Although, more recent studies have suggested variability that is due to environmental factors; additional data processing [14,43] or differing methods of data collection [26,43] can increase data comparability. However, it is not clear whether this would be the case for all external variables. 

In summary, costs of IRT devices of similar quality to those tested in the academic literature are prohibitively expensive to the adoption of IRT; this research aims to determine whether, as technology has advanced, LCDs are a viable option for use as a lameness detection aid.

## 2. Materials and Methods

### 2.1. Experiment Design

#### 2.1.1. Data Collection

This study was carried out in March–April 2022; ethical approval was obtained from the RVC-CRERB (CR2021-020-2). Data were collected from a dairy herd of 83 Holstein Friesian cows milked in a tandem parlour in Hertfordshire. Two IRT devices were used to capture the thermal images—FLIR T620bx (TELEDYNE FLIR, West Malling, UK) and CAT s62-Pro Smartphone (CAT Phones, Reading, UK). Key specifications are documented in Table 1.

**Figure 1:**
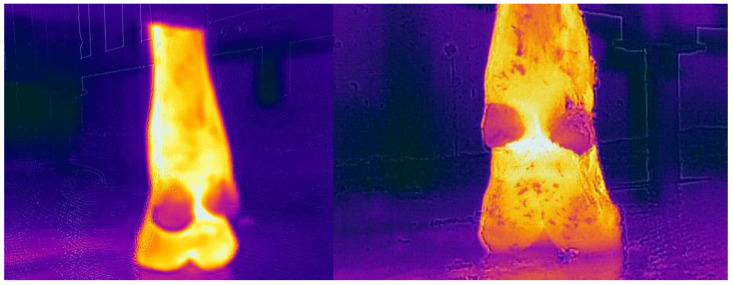
(**Left**) A thermogram captured by the CAT s62 Pro Smartphone of the target area of imaging for all images taken in this study. The target area assessed is the point of hottest temperature present below the heel bulbs; this thermogram was cropped from a portrait image. (**Right**) A thermogram captured by the FLIR T620bx of the target area of imaging for all images taken in this study. The target area assessed is the point of hottest temperature present below the heel bulbs.

Ambient temperature and humidity were measured using a PT6508 hygrometer (Protmex, Shenzhen, China); at each data collection event, IRT devices were calibrated accordingly. Thermograms of the target area of each cow were captured from 0.5 m during morning milking with both cameras; the target area was that of the heel bulbs and below in the hind feet (Figure 1). Emissivity was set at 0.95 and the reflected temperature was 20 °C. 

Cattle feet were not washed or cleaned before the images were taken, as this decreases effectiveness of IRT devices in lameness detection [24,46,49] and would hinder the assessment of the effectiveness of the LCD within the milking parlour. Upon exit of the milking parlour, each cow’s MS was recorded using the AHDB four-point scale (Table 2) and the details of the affected leg(s) were recorded. Data collection was repeated on four occasions over a 2-week period.

#### 2.1.2. Data Processing

**Figure 2:**
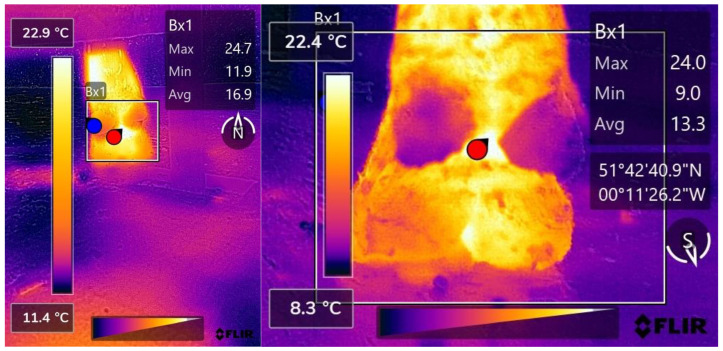
(**Left**) A thermogram captured by the CAT s62 Pro Smartphone and processed with FLIR Thermal Studios showing the point of maximum temperature within Bx1, labelled by the red marker, recorded as being caudal to the heel-bulbs on the medial side of the medial claw. The maximum temperature value transferred for statistical analysis from this thermogram was 24.7 °C. (**Right**) A thermogram captured by the FLIR T620bx and processed with FLIR Thermal Studios showing the point of maximum temperature within Bx1, labelled by the red marker between the heel bulbs. The maximum temperature value transferred for statistical analysis from this thermogram was 24.0 °C.

**Figure 3:**
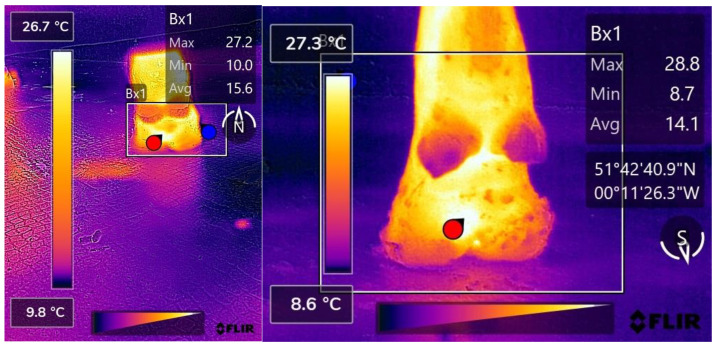
(**Left**) A thermogram captured by the CAT s62 Pro Smartphone and processed with FLIR Thermal Studios showing the point of maximum temperature within Bx1, labelled by the red marker, recorded as being caudal to the heel-bulbs on the medial side of the medial claw. The maximum temperature value transferred for statistical analysis from this thermogram was 27.2 °C. (**Right**) A thermogram captured by the FLIR T620bx and processed with FLIR Thermal Studios showing the point of maximum temperature within Bx1, labelled by the red marker, recorded as being caudal to the heel-bulbs on the medial side of the medial claw. The maximum temperature value transferred for statistical analysis from this thermogram was 28.8 °C.

A total of 332 pairs of hind foot thermograms were obtained across the four data collection days; of these, 11 pairs when analysed were not of adequate quality for use in this study; this was due to the target area not being fully present within the thermograms. These data were collected for both the LCD and the high-cost IRT device (HCD); 203 pairs were from sound cattle and 118 pairs from lame cattle. Thermograms obtained from both the HCD and LCD were manually analysed using FLIR Thermal Studios to determine the maximum temperature of each foot (Figure 2). If, on analysis of the thermogram, the point of maximum temperature was not between the heel bulbs, as was the ordinary finding, the positioning was recorded; Figure 3 shows an example where the area of maximum temperature was caudal to the heel bulbs on the medial side of the lateral claw. Images were not qualitatively analysed in this study, so temperature scales were not adjusted. 

### 2.2. Statistical Analysis

The maximum temperature of each thermogram was transferred to Microsoft Excel (Microsoft, Washington, DC, USA) and matched to the MS of the cow it was taken from; cows with MS scores of 2 and 3 were classified as lame. Statistical analysis was carried out in GraphPad Prism 9 (GraphPad Software, San Diego, CA, USA).

#### 2.2.1. Environmental Factor Analysis

All data, grouped by day of collection and device used, were assessed for normality using the D’Agostino and Pearson test. Not all groups were normally distributed and a Kruskal Wallis test with Dunn’s multiple comparison was used to assess the significance. 

To account for differences in environmental factors between data collection days, a temperature adjustment method was used, similar to those used in previous literature [43,44]. The average maximum foot temperature of sound cattle was calculated for each data collection day for each IRT device. Residuals between this value and each data point in the corresponding group were calculated. The set of residual data, grouped by data collection day and device used, was assessed by a Kruskal Wallis test with Dunn’s multiple comparison.

#### 2.2.2. Adjusted Temperature Analysis

Using the MS, the residual data was split into three groups for each IRT device: non-lame cows’ feet; the identified lame foot of the cow; and the healthy foot of a lame cow. A Kruskal Wallis test with Dunn’s multiple comparison was carried out on these. Each group obtained by the LCD was compared against the corresponding group collected by the HCD, using Dunn’s multiple comparison test.

#### 2.2.3. Temperature Difference Analysis

The difference between temperatures of individuals’ hind feet were calculated and sorted into groups dependent on MS, for two separate analyses; MS: 0, 1, 2, and 3 for one analysis, and for a second analysis, MS 0 and 1 were grouped, and MS 2 and 3 were grouped—all these were grouped separately for each IRT device. A Kruskal Wallis test with Dunn’s multiple comparison was carried out for both analyses and corresponding groups obtained from each IRT device were assessed. 

#### 2.2.4. Threshold Analysis

To compare quantitatively to the previous literature and calculate potential efficacy of the LCD, threshold values for lameness determination were calculated. A receiver operating characteristic (ROC) graph was created and analysed to determine the sensitivity and specificity for both IRT devices with both the method of residual adjusted temperature and temperature difference [43,51]; positive and negative predictive values were calculated for each threshold value.

## 3. Results

### 3.1. Environmental Factor Results

**Figure 4:**
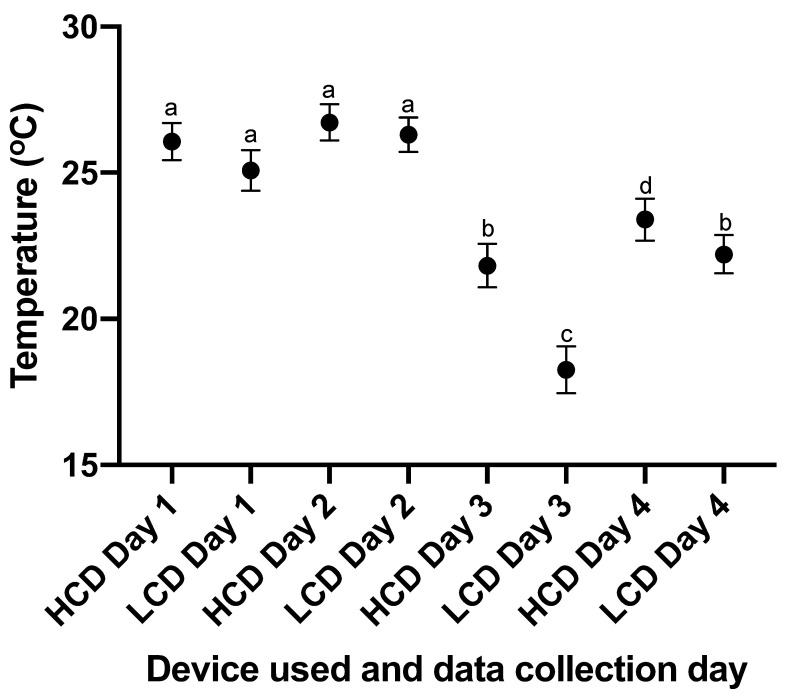
A graph showing the mean and 95% confidence interval of the maximum temperature of all cattle feet across each data collection day (n = 160, 164, 166, and 158 for data collection from each device on days 1–4, respectively) collected by the CAT S62-Pro Smartphone (LCD) and the FLIR T620bx (HCD). ^abcd^ different superscripts indicate significant differences between means at *p* < 0.05.

The results of the environmental factor analysis show a significant difference between average foot temperatures obtained on each data collection day (Figure 4). Ambient temperature, humidity, mean, and standard deviation of healthy feet obtained on each collection day are shown in Table 3.

### 3.2. Adjusted Temperature Results

**Figure 5:**
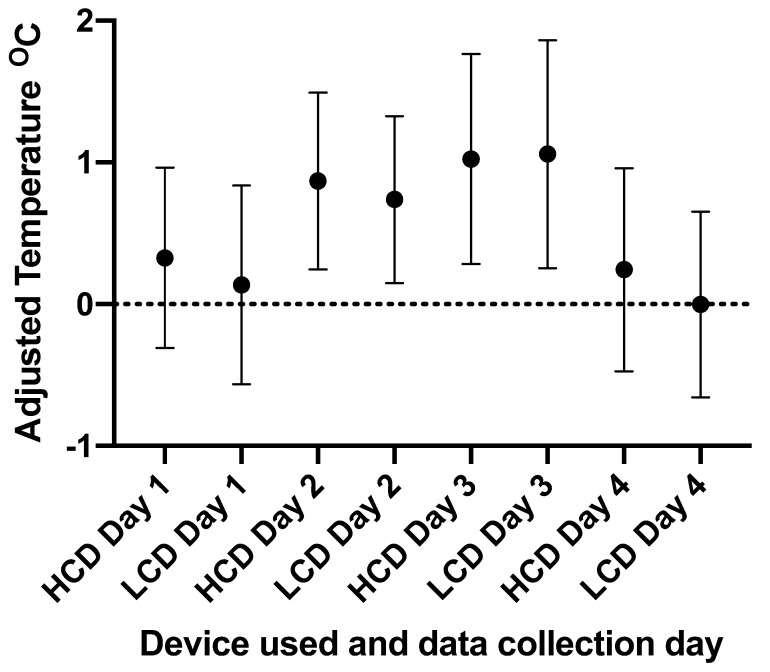
A graph showing the mean and 95% confidence interval of the temperature of cattle feet, once adjusted for ambient temperature, across each data collection day (n = 160, 164, 166, and 158 for data collection from each device on days 1–4, respectively) collected by the CAT S62-Pro Smartphone (LCD) and FLIR T620bx (HCD). The adjusted temperatures were calculated from the residuals from the average healthy foot temperature on each data collection day. The dotted line at 0 represents foot temperatures equivalent to the average temperature of healthy feet for the corresponding day of data collection. No data groups were statistically significant from each other.

The results of the residual temperature data analysed show no significant difference between the median residual foot temperatures obtained on each day of data collection (Figure 5); this lack of a significant difference is true for both the LCD and the HCD. 

**Figure 6:**
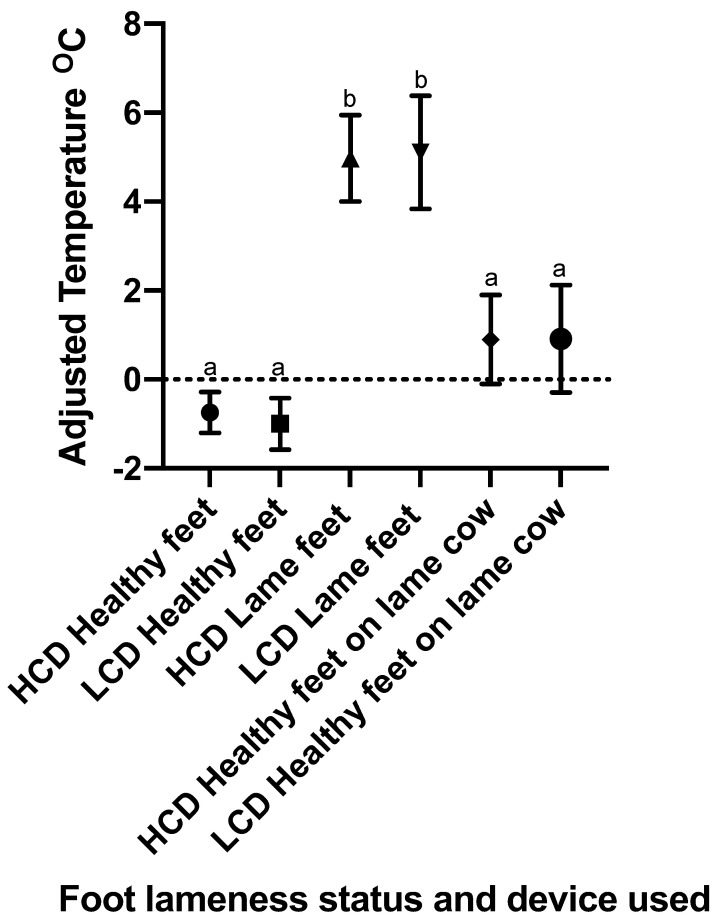
A graph showing the mean and 95% confidence interval of adjusted temperatures for the healthy cattle feet, lame cattle feet, and the non-lame feet present on a lame cow (n = 406, 121, and 115 for each group, respectively; both devices had an equivalent n in corresponding groups). The adjusted temperatures were calculated from the residuals from the mean healthy foot temperature on each data collection day. This graph shows both the data set obtained from the CAT S62-Pro Smartphone (LCD) and that from the FLIR T620bx (HCD). The dotted line at 0 represents foot temperatures equivalent to the mean temperature of healthy feet for the corresponding day of data collection. ^ab^ different superscripts indicate significant differences between means at *p* < 0.05.

Significant differences were identified between the groups of ‘healthy feet’ and ‘lame feet’ and between groups of ‘lame feet’ and ‘healthy feet from lame animals’ for both IRT devices used in this study when residual temperature data were assessed (Figure 6). No other comparisons, including those comparisons between equivalent groups measured by differing IRT devices, had a significant difference.

### 3.3. Temperature Difference Results

**Figure 7:**
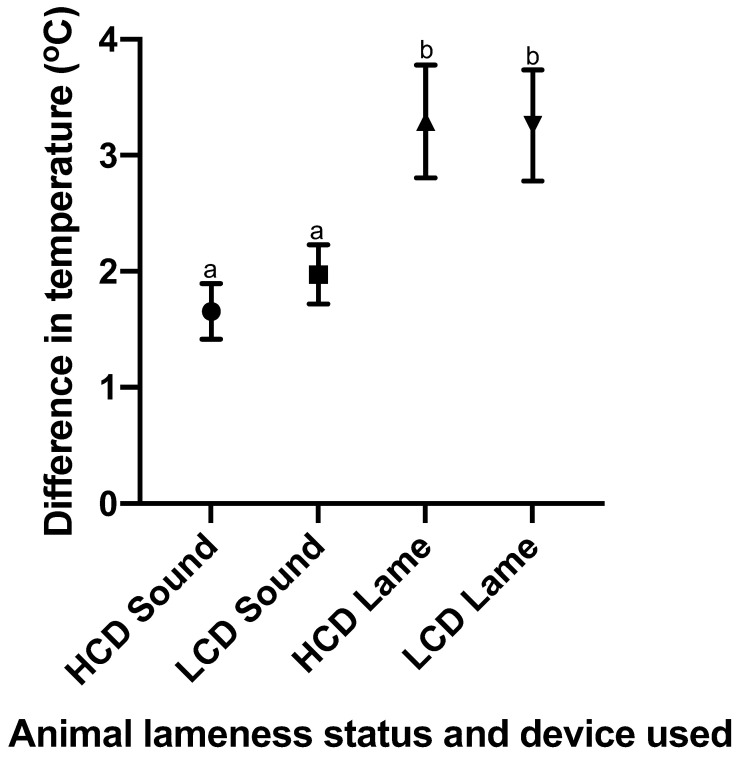
A graph showing the mean and 95% confidence interval of the temperature difference between the hind feet of each individual cow, for the categories of lame and sound cow (n = 203 and 118, respectively; both the devices used had an equivalent n in corresponding groups); these were determined by mobility scores of 0 and 1 being classed as sound, and mobility scores of 2 and 3 being classified as lame. This graph shows both the data set obtained from the CAT S62-Pro Smartphone (LCD) and that from the FLIR T620bx (HCD). ^ab^ different superscripts indicate significant differences between means at *p* < 0.05.

Significant differences were identified between lame and sound cattle upon analysis of the temperature difference between hind feet for both IRT devices (Figure 7). There is no significant difference present between equivalent groups measured by differing IRT devices. 

**Figure 8:**
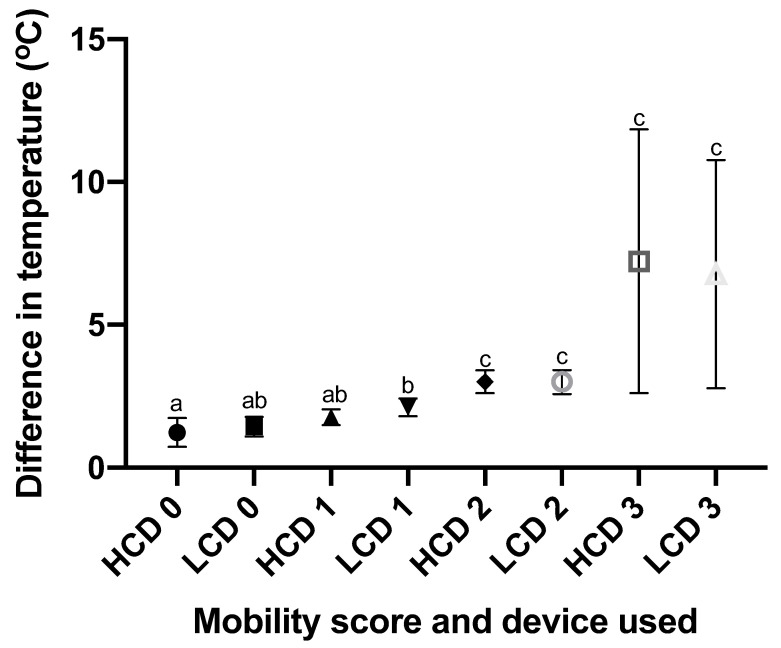
A graph showing the mean and 95% confidence interval of temperature difference between the hind feet of each individual cow, for each AHDB mobility score 0, 1, 2, and 3 (n = 43, 160, 110, and 8, respectively; both devices had an equivalent n in corresponding groups). This graph shows both the data set obtained from the CAT S62-Pro Smartphone (LCD) and that from the FLIR T620bx (HCD). ^abc^ different superscripts indicate significant differences between means at *p* < 0.05.

Significant differences were identified between the groups of MS 1 and 2 for both devices on analysis of hind feet temperature difference data (Figure 8). No other comparisons resulted in significant differences including those between equivalent groups measured by differing IRT devices.

### 3.4. Threshold Results

**Figure 9:**
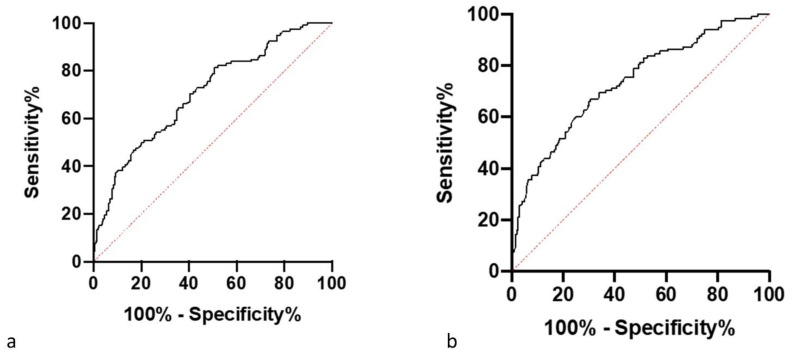
(**a**) An ROC curve showing the sensitivity and specificity of different threshold values in determining whether a cow is lame based on the data collected on the maximum ambient adjusted temperature calculated from the residuals from the average healthy foot temperature on each data collection day collected by the CAT s62-Pro Smartphone (LCD). (**b**) An ROC curve showing the sensitivity and specificity of different threshold values in determining whether a cow is lame based on the data collected on the maximum ambient adjusted temperature calculated from the residuals from the average healthy foot temperature on each data collection day collected by the FLIR T620bx (HCD).

**Figure 10:**
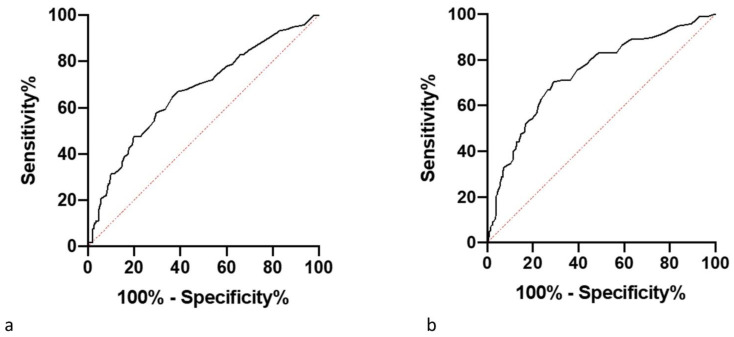
(**a**) An ROC curve showing the sensitivity and specificity of different threshold values in determining whether a cow is lame based on the data collected of the difference between the hind foot temperatures, calculated from the residuals from the average healthy foot temperature on each data collection day collected by the CAT s62-Pro Smartphone (LCD). (**b**) An ROC curve showing the sensitivity and specificity of different threshold values in determining whether a cow is lame based on the data collected of the difference between the hind foot temperatures, calculated from the residuals from the average healthy foot temperature on each data collection day collected by the FLIR T620bx (HCD).

Figure 9 and Figure 10 show receiver operating characteristic (ROC) curves for the threshold values set for the difference in hind foot temperature and maximum adjusted residual temperature of a foot. Optimum threshold values for each method are the same for both devices. The HCD had greater sensitivity and specificity values for both methods compared with the LCD. The HCD has higher sensitivity and specificity for the method of involving hind feet temperature difference, conversely for the LCD, the maximum adjusted residual temperature method had the greater specificity and the hind feet temperature difference method had the greater sensitivity (Table 4).

## 4. Discussion

### 4.1. Findings

#### 4.1.1. Environmental Factor Findings

The significant difference between the average foot temperatures on each data collection day, for both the LCDs and HCDs, and the lack of significance after residual adjustment suggests that uncontrolled environmental factors affect the normal temperature of cattle. This finding is similar to previous research that suggests that, although environmental factors are uncontrollable, they can be accounted for by statistical adjustment [21,26,39,41,52,53,54,55]. The key uncontrolled environmental factor cannot be confidently determined. However, it is likely that ambient temperature is the predominant factor as on collection days when temperature was equal and other factors affecting foot temperature, such as humidity, windspeed, and foot debris, were different [42]; there was no statistically significance between average foot temperatures. Previous investigations studying IRT in foot and mouth disease diagnosis came to similar conclusions [54]; to solve this issue, a table of normal foot temperature values at different ambient temperatures was created [53,54]. If a similar approach were taken for lame cattle, this may remove the need for the large sample sizes and data processing reported to be prohibitive to IRT adoption [32]. Additionally, this suggests ambient temperature could be the reason for the previously found lack of inter-farm comparability [46], as, if not accounted for, there will be significant variation in IRT-detected temperature. It may be worth repeating previous approaches with consideration for ambient temperature to determine if inter-farm comparison becomes possible.

#### 4.1.2. Adjusted Temperature Findings

The significant difference between the maximum adjusted temperature detected by the LCD between healthy and lame feet indicates it can detect lame animals. Moreover, the significance between the LCD groups of ‘lame feet’ and ‘healthy feet on lame cattle’ show that the lame limb can be detected, a key criteria of lameness detection systems [2]. The lack of statistical significance between the two devices corroborates this, suggesting the LCD is as effective as the HCD as a detection aid; similar to devices previously shown to be advantageous in this use [17,24,26,43]. However, the larger LCD confidence interval for the group of ‘lame limb’, compared with the HCD, suggests the LCD is less accurate and reliable. This is likely because of the lower specifications of the LCD, particularly the lower resolution. Previous research in other fields has found this often results in reduced thermogram detail and greater potential for erroneous maximum temperature values [32,42]; these devices previously tested had a marked drop in maximum temperature recorded at an increasing distance from the target; the magnitude of the drop correlated with resolution of the device used [42]. Despite the lack of HCDs used in this previous research, it is likely that this pattern is true for data in this current study, which suggests the distance used in this study may be sub-optimal for the LCD, as closer distances may provide more reliable results. Similar to previous research [43], there is no significance between sound limbs from lame cows and limbs from sound cows, despite the visual increase in temperature limbs on lame cattle. This is not supported by the mechanism of supportive limb lameness found in horses [28]. However, similar to previous research [43] with a larger sample size and greater statistical power, significance may be reached. Although academically interesting, this measurement will not affect IRT effectiveness as it is not of relevance to a farmer in lameness detection.

#### 4.1.3. Temperature Difference Findings

The significance found between lame and sound cattle on the assessment of hindlimb temperature difference supports previous findings [43] that this is a suitable method for lameness detection and that no further data processing is required to account for environmental factors [54]. However, neither IRT device could distinguish between MS of 0/1 or 2/3, suggesting that IRT temperature does not have an exact correlation with lameness severity; previous research suggests that this is likely due to either the subjectivity of MS classification or from the type of lesion present, with lesions such as DD causing an increased level of inflammation compared with others; altering the method to involve lesion identification through lifting of the foot would prevent these issues [19,20,25,56]. Regardless, this does not majorly alter the effectiveness of IRT, as it is recommended to only intervene for animals of MS 2 and above [50]. Akin to the case of the previously discussed method, with this temperature difference method, the LCD and HCD are comparable owing to the lack of significant difference between any paired comparisons. However, it is interesting to note that, though not statistically significant, the HCD had a higher mean for lame cattle and a lower mean for sound cattle than the LCD. This larger interval may enable more accurate lameness scoring decisions to be made by the HCD.

#### 4.1.4. Threshold Findings

Although there are no significant differences between the IRT devices, the calculated threshold values show that the LCD is less effective; again, this might be explained by the lower specifications, resulting in a greater spread of data, or a lack of optimum operation. Additionally, although there is significant difference between lame and non-lame animals, the LCD has not reached sensitivity and specificity values close to those in the literature; previously, a maximum of 89.1% sensitivity [38] and 87.7% specificity [53] has been reached. However, as the HCD also has reduced values, this discrepancy is likely due to methodology or prevalence of lesions on each individual farm, as there is little difference in effectiveness shown between the methods used in this study; the most critical difference is likely the criterion used to diagnose an animal as being lame. Previously, cattle feet were lifted to detect foot lesions [23,24,26,46], allowing an increased number of lame animals to be detected than in MS assessment. Additionally, factors associated with data collection during routine milking may have reduced effectiveness. Regardless, although less effective, the LCD has potential use as a lameness detection aid, especially as MS is nonapplicable in the milking parlour. LCD use may be more effective if used to rule out lameness due to the low PPV and higher NPV, a finding supported by previous use of infrared thermometers [44]. This may enable more targeted cattle treatment as, often, only certain cows are selected for routine foot trimming; the LCD could be used to aid these decisions. It may be beneficial to use the method of temperature difference as the lack of additional processing required to account for external factors, previously described [54], is a benefit that does not affect effectiveness. However some previous research disagrees, finding this to be a less effective method, especially with bilaterally lame cattle [43]; on farms with high levels of MS 3 cattle, this method might be less precise. 

### 4.2. Implications

Combined with previous research [21,26,39,41,52,53,54,55], the findings from this study show IRT and the LCD has potential use as a lameness detection aid and that the LCD has similar effectiveness to the HCD. However, it is not currently a viable replacement for mobility scoring, instead likely a useful adjunct. To completely assess LCD cost-effectiveness, multi-farm investigations would be required; individual farm effectiveness will likely depend on the current systems used and the existing skill of workers, although it is likely that, in all herds, some benefits can be realized owing to ease of use of the LCD [57].

**Figure A1:**
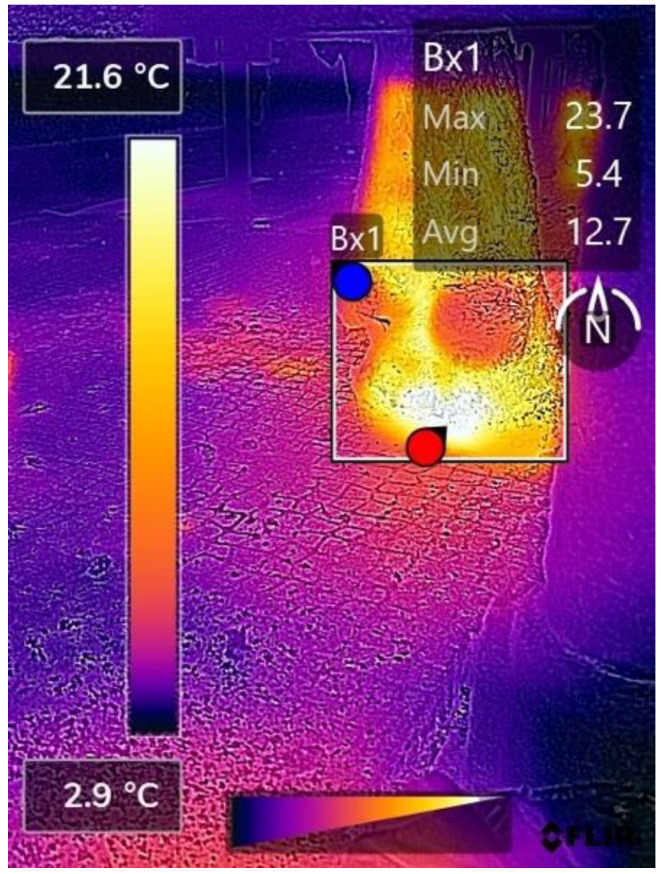
A thermogram, obtained by the CAT s62-Pro Smartphone (LCD), of cattle diagnosed with digital dermatitis on routine foot trimming, displaying a pattern of heat with maximum intensity over the interdigital space, rather than between the heel bulbs.

**Figure A2:**
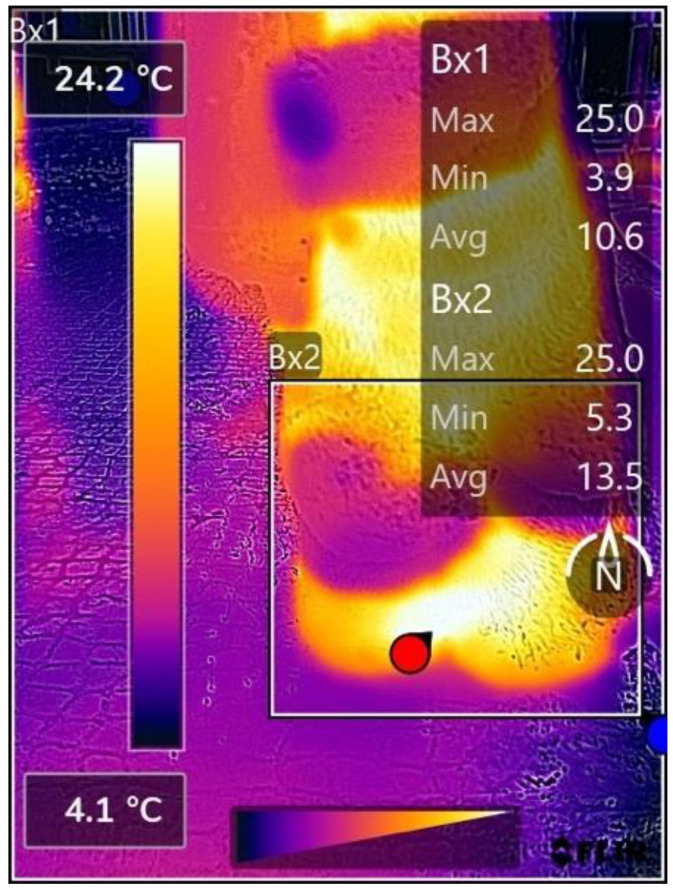
A thermogram, obtained by the CAT s62-Pro Smartphone (LCD), of cattle diagnosed with digital dermatitis on routine foot trimming, displaying a pattern of heat with maximum intensity over the interdigital space, rather than between the heel bulbs.

**Figure A3:**
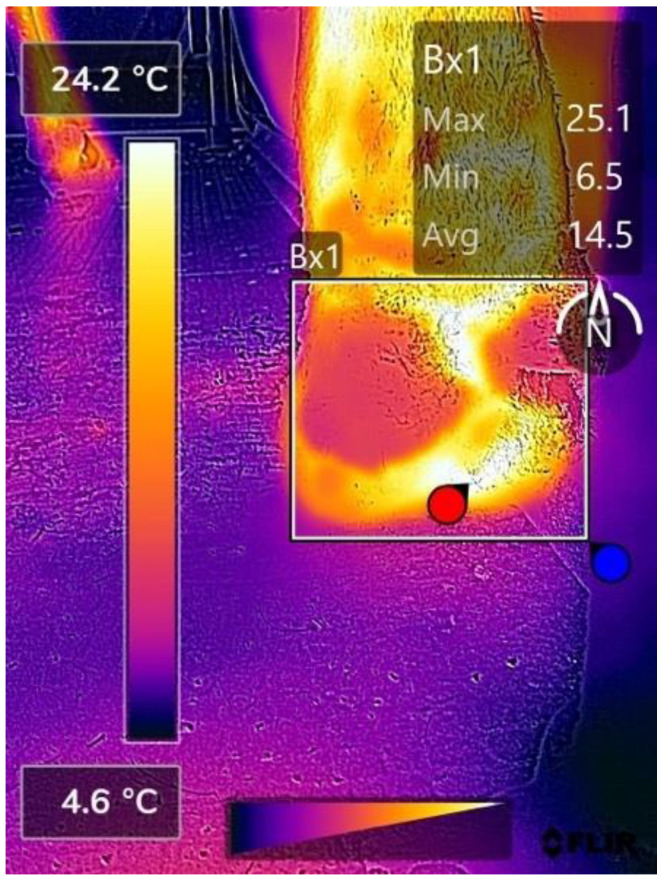
A thermogram, obtained by the CAT s62-Pro Smartphone (LCD), of cattle diagnosed with digital dermatitis on routine foot trimming, displaying a pattern of heat with maximum intensity over the interdigital space, rather than between the heel bulbs.

**Figure A4:**
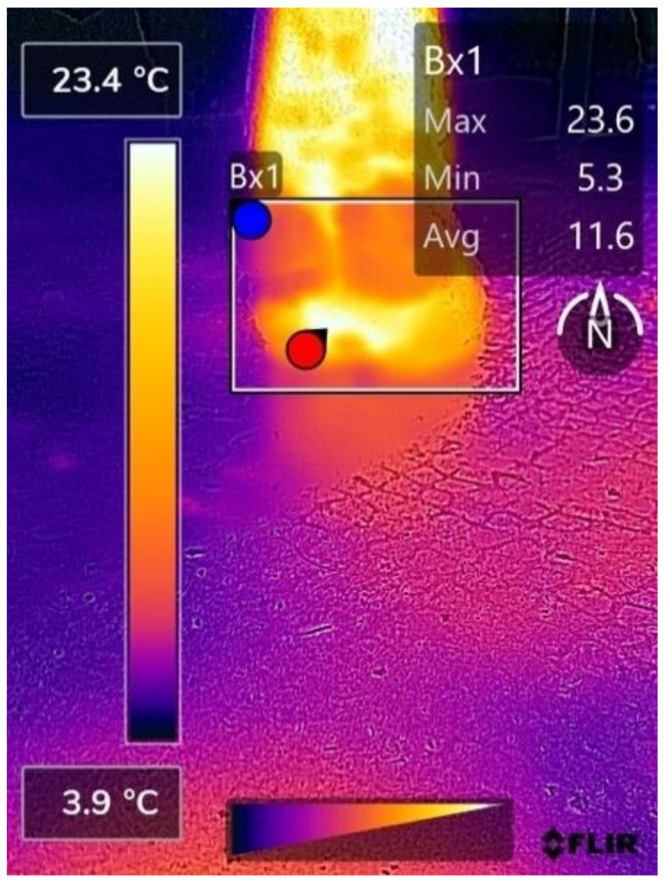
A thermogram, obtained by the CAT s62-Pro Smartphone (LCD), of cattle diagnosed with digital dermatitis on routine foot trimming, displaying a pattern of heat with maximum intensity over the interdigital space, rather than between the heel bulbs.

**Figure A5:**
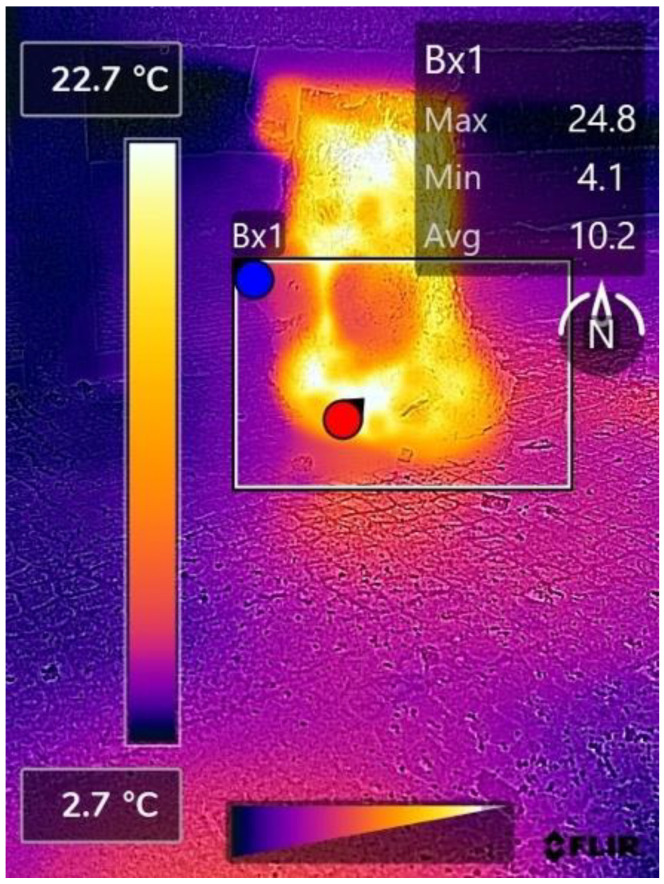
A thermogram, obtained by the CAT s62-Pro Smartphone (LCD), of cattle diagnosed with digital dermatitis on routine foot trimming, displaying a pattern of heat with maximum intensity over the interdigital space, rather than between the heel bulbs.

**Figure A6:**
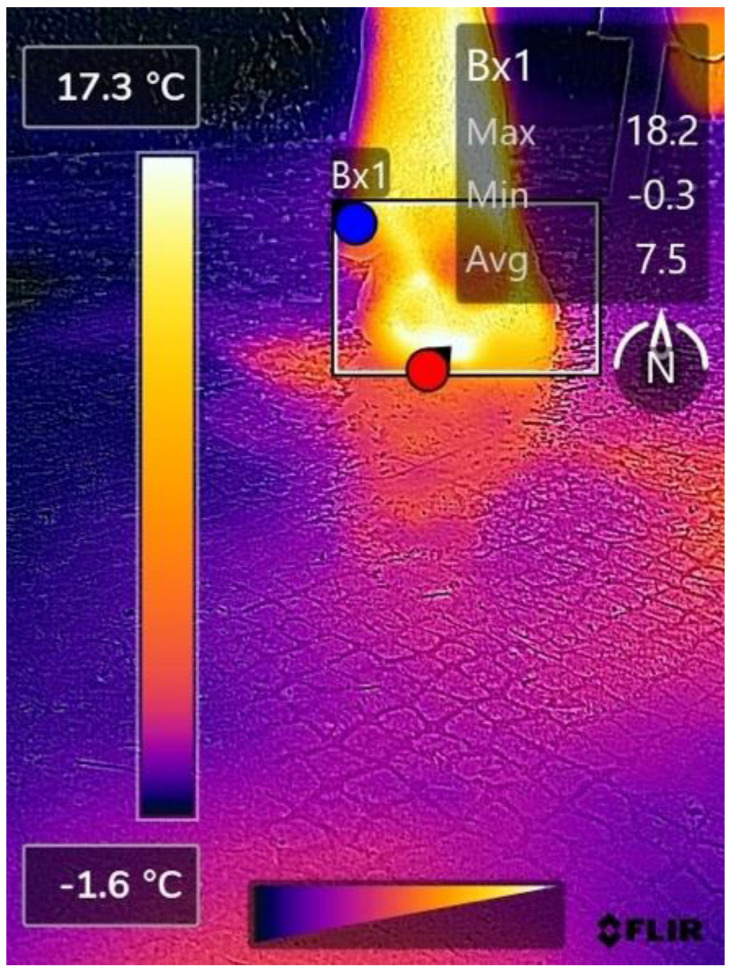
A thermogram, obtained by the CAT s62-Pro Smartphone (LCD), of cattle diagnosed with digital dermatitis on routine foot trimming, displaying a pattern of heat with maximum intensity over the interdigital space, rather than between the heel bulbs.

IRT devices have some ability to determine the lesion present when a cow is categorised as lame [3,15,24,46], although investigation of this was not an aim of the study. Despite this, after IRT data collection, there was a routine foot trimming. Anecdotally, the data obtained show a pattern between the position of the point of maximum temperature and the type of lesion present; thermograms with the point maximum temperature positioned lower than the heel bulbs appear to correlate with a diagnosis of DD as those cattle with identified active DD lesions had this pattern present on their thermograms, Figure A1, Figure A2, Figure A3, Figure A4, Figure A5 and Figure A6. This is likely because of the marked increase in interdigital skin temperature in active DD lesions, found in previous research [14]. Reliable conclusions cannot be drawn from this because no controlled test was undertaken; more research is needed in this area to determine both if LCDs can determine lesion type and whether this temperature pattern is pathognomonic for DD. 

The congruence of optimum threshold values of the IRT devices suggests that there is an exact temperature, dependent on external factors, for when a cow is lame. This suggests that there is inter-IRT device repeatability and that the cost of the device only determines the accuracy of recorded IRT temperature; a general threshold for lameness detection could be set for any IRT device using the same method of data collection. This cannot be corroborated, as previous research has used differing methodologies [14,15,24,25,37,38,41,46], but this should be investigated further as it may result in IRT being used for nationwide assessment [32]. However, it should be noted that an optimum method should be identified, and currently it is unclear what this would be [15,43]. 

### 4.3. Limitations

There are limitations to this study that should be acknowledged. MS is a subjective measure of lameness, and in future studies, it may be beneficial to have multiple mobility scorers [58] or, to identify a greater proportion of animals with foot lesions, feet could be lifted for assessment. Additionally, this study had a small sample population with a relatively low level of cattle of MS 3; it would be beneficial to expand the study to encompass a greater population of lame cattle across multiple farms. 

### 4.4. Further Research

This study has highlighted some areas in which future research may be beneficial. Corroboration is needed to determine if threshold values remain unchanged for equivalent methodologies on multiple farms, as well as to determine if inter-farm comparison and use in the national herd is possible. Additionally, the effectiveness of lesion identification of the LCD should be assessed through the lifting of feet, in order to determine if the anecdotal account is true and if the HCD offer any benefits in this method of use. 

## 5. Conclusions

To conclude, this study shows that LCDs can be used as a lameness detection aid; however, environmental factors hinder repeatability of data for all IRT devices, and thus need to be accounted for; both methods of accounting for environmental factors in this study were acceptable. The minimal differences between the HCD and LCD suggest that LCDs could be used as an on-farm alternative to HCDs, although the sensitivity of the LCD is low, appearing to be more effective at detecting those that are not lame. LCDs have the potential for widespread application as differing IRT devices do not affect optimum threshold values; there is possibility that LCDs could be used as an objective, cost-effective method of assessing the lameness of the national herd, which may prove a useful adjunct to the current lameness detection methods. 

## Figures and Tables

**Table 1:** Thermal resolution, sensitivity, range, accuracy, and approximate cost of both the CAT s62 Pro Smartphone (LCD) and the FLIR T620bx (HCD).

Camera	Resolution (Pixels)	Thermal Sensitivity (°C)	Temperature Range (°C)	Accuracy	Approximate Cost
CAT s62-Pro Smartphone	160 × 120	<0.05	−20 to 400	3% or 3 °C	GBP 400
FLIR T620bx	640 × 480	<0.04	−40 to 650	2% or 2 °C	GBP 20,000

Source: [47,48].

**Table 2:** The AHDB mobility scoring criteria and suggested response for each grade, 0–3. Grades of 2 and 3 are classified as lame cows.

AHDB Lameness Score	Criteria	Suggested Response
0	Locomotion with smooth and long steps. Weightbearing evenly across all 4 feet. The back is flat	Routine trim if needed
1	Uneven rhythm to gait or uneven weightbearing Shortened stride-length The affected limb is not identifiable	Routine trim when needed
2	Uneven weight bearing on an identifiable limb Shortened stride-length Arched back	Lift the foot to identify the issue as soon as possible
3	Very lame Unable to maintain pace speed of a human walking May be limping	Urgent medical attention needed; the animal should not be made to walk.

Source: [50].

**Table 3:** The ambient temperature, relative humidity, mean foot temperature, and standard deviation of temperature of sound hind feet on each of the data collection days for both infrared thermography devices used in this study.

Data Collection Day	Ambient Temperature (°C)	Humidity (%)	LCD: Mean and Standard Deviation (±°C)	HCD: Mean and Standard Deviation (±°C)
1	10	61	25.09 ± 4.479	26.07 ± 4.074
2	10	86	26.31 ± 3.819	26.72 ± 4.054
3	2	83	18.26 ± 5.249	21.82 ± 4.825
4	5	90	22.21 ± 4.089	23.40 ± 4.462

**Table 4:** Calculated sensitivity, specificity, and positive predictive and negative predictive values from optimum thresholds calculated using an ROC curve using maximum adjusted temperature of a cattle foot, calculated from the residuals from the average healthy foot temperature on each data collection day, and the temperature difference between the hind limbs of a cow, for both the CAT s62-Pro Smartphone (LCD) and the FLIR T620bx (HCD).

Device Used	Test	Threshold Value (°C)	Sensitivity (%)	Specificity (%)	Positive Predictive value (PPV) (%)	Negative Predictive Value (NPV) (%)
LCD	Maximum adjusted temperature	2.40	64.41	64.53	51.35	75.72
HCD	Maximum adjusted temperature	2.40	69.49	66.01	54.30	78.82
LCD	Temperature difference	1.85	66.95	61.08	50.00	76.07
HCD	Temperature difference	1.85	70.34	70.94	58.45	80.45

## Data Availability

The data presented in this study are available on request from the corresponding author.
